# A Brief Body-Mind-Spirit Group Therapy for Chinese Medicine Stagnation Syndrome: A Randomized Controlled Trial

**DOI:** 10.1155/2018/8153637

**Published:** 2018-06-14

**Authors:** Siu-man Ng, Lingli Leng, Rainbow T. H. Ho, Zhangjin Zhang, Qi Wang

**Affiliations:** ^1^Department of Social Work and Social Administration, The University of Hong Kong, Hong Kong; ^2^Centre on Behavioral Health, The University of Hong Kong, Hong Kong; ^3^School of Chinese Medicine, The University of Hong Kong, Hong Kong

## Abstract

**Background:**

Stagnation syndrome, a diagnostic entity in traditional Chinese medicine (TCM), is characterized by mind-body obstruction-like symptoms. Although TCM has long-established symptom-relief treatments, a comprehensive mind-body intervention was called for.

**Purpose:**

The study evaluated the efficacy of a six-session body-mind-spirit (BMS) group therapy for persons with stagnation syndrome.

**Method:**

A 2-arm randomized controlled trial design was adopted. The control group received a parallel general TCM instruction course. Both groups completed a pretest (T0), posttest (T1), and 2-month follow-up assessment (T2). The measures included self-report scales on stagnation, depression, anxiety, physical distress, daily functioning, and positive and negative affect; the other measure was of salivary cortisol, a biological marker of stress.

**Results:**

Data on 111 adults with stagnation syndrome were included in the analysis. Completion rates were high (over 87%) for both the intervention and control groups. Repeated-measures multivariate MANOVA revealed a significant combined effect with large effect size (eta-squared = 0.42). Repeated-measures ANOVA further revealed that the intervention group showed significant improvements in stagnation, the primary outcome, with medium effect size (eta-squared = 0.11). The intervention group also showed significant improvements in depression, physical distress, everyday functioning, and negative affect (eta-squared = 0.06 to 0.13). Post hoc analysis revealed that the intervention group showed significant improvements over the control group in cortisol level at 2-month follow-up assessment (T0 versus T2) with small effect size (eta-squared = 0.05), but not at posttest (T0 versus T1).

**Conclusions:**

Overall, the findings indicate that our brief BMS group therapy intervention for stagnation syndrome is efficacious. Moreover, the intervention resulted in a number of substantial improvements in the physical and mental health domains.

## 1. Introduction

Stagnation syndrome, a long-established diagnostic entity of traditional Chinese medicine (TCM), is a prevalent complaint in both primary care settings and general population, categorized under internal medicine. Conceptualized as a set of tightly connected mind-body health condition, it is characterized by a cluster of somatic symptoms, such as feeling clogged at throat or chest, fatigue, sleep disturbances, and bowel dysfunctions. From the Western medicine perspective, stagnation syndrome can be understood as a somatic symptom disorder, featured by multiple functional somatic symptoms that are psychosomatic in essence [1]. Moreover, stagnation syndrome is often a chronic condition and can significantly impair one's physical and mental health. Ng and colleagues conducted a survey of stagnation syndrome among a random community sample of 755 adults in Hong Kong and estimated the point prevalence of the disorder at 6.2%. Compared with healthy counterparts, adults with stagnation syndrome reported significantly higher level of physical distress, depression, and anxiety symptoms [2]. Consistently, it was also reported that stagnation patients experienced considerably worsened physical and mental health condition than general population, particularly featured by heightened physical distress and debilitating daily functioning [1].

The clinical presentation and etiology of stagnation syndrome have long been explicated in the earliest TCM classic,* Inner Canon of the Yellow Emperor* [3]. Repression of emotions, particularly of anger, is the very first step in the formation of the syndrome. For example, a person may often feel offended but be unable to vent his or her anger or resentment because he or she feels obliged to behave in a socially acceptable manner embedded in particular social contexts. If such repression of emotions is severe and prolonged, the person's physical health will be affected. According to TCM's five-element theory, repressed anger affects the free flow of qi (vital energy; also known as chi) in the liver meridian system, resulting in “liver qi stagnation” and a corresponding cluster of symptoms; these include sleep disturbances, dizziness, headaches, fatigue, chest pains, sexual dysfunction, and (for adult women) irregular menstruation. Furthermore, again according to TCM's five-element theory, liver qi stagnation affects the spleen meridian system, resulting in another cluster of obstruction-like symptoms; these include feeling that something indescribable is trapped inside one's head, throat, or chest; feeling that food is clogged inside one's stomach; experiencing bowel dysfunctions; and experiencing abdominal distension. As the physical, emotional, and behavioral symptoms reinforce each other, stagnation syndrome is often a chronic condition. Most people with stagnation syndrome adopt self-regulation/inhibition and struggle to maintain productive roles at home and in the workplace, wrestling with persistent mind-body discomfort.

Consistently, a number of Western medical researchers have suggested that habitual repression of emotions may impair the immune function and cause various mental and physical problems [4–6]. Based on a series of experimental studies, Pennebaker proposed that inhibition of thoughts and feelings intensifies internal stress responses (Consedine, Magai & Bonanno, 2003) [7, 8]. A consequence of sustained physiological stress responses is an elevated cortisol level [9, 10]. If prolonged, the inhibition can lead to increased risk of immunological dysfunction, mood disorders, headaches, hypertension, and other general health problems [11–15].

Although stagnation syndrome is uniquely documented in Chinese culture, the disorder is also found in other civilized societies. In the US, stagnation syndrome is one of the most common maladies TCM practitioners deal with [16]. In the UK, data from TCM teaching clinics showed that 27% of all herbal medicine prescriptions included various modifications of the classic “rambling powder”, a core, long-established herbal medicinal formula for treating stagnation syndrome [17].

In modern medical science, stagnation syndrome is often compared with depression [16]. The clinical presentations of stagnation syndrome comprise both emotional/behavioral inhibitions and obstruction-like physical symptoms, which are also common in depression. Stagnation syndrome used to be considered the TCM counterpart of Western medicine's diagnostic entity of depression [18]. Previous studies have shown that stagnation syndrome has a significant positive correlation with depression and anxiety [19, 20]. Although there is some degree of overlapping, stagnation syndrome and depression are distinct syndromes distinguishable in their conceptualizations, clinical presentations, and patient profiles. “Stagnation” literally means “not flowing, clogged”; the state of depression is characterized by dejection and hopelessness. Categorized under internal medicine, stagnation has a more prominent set of somatic symptoms, while symptoms of depression are more related to changes in mood, cognition, and behavior. Depression is categorized as a psychiatric problem; stagnation syndrome is not. In addition, unlike those who suffer from depression, people with stagnation syndrome exhibit no significant gender-related differences; and they tend to be well-educated younger adults with professional or managerial positions [2]. Because those with stagnation syndrome are not stigmatized as having a mental disorder, Chinese people are open about suffering from the syndrome and do not hesitate to seek treatment for it.

To make the construct useful for mental health practitioners, Ng and colleagues operationalized the stagnation syndrome construct by developing a scale in a study of 602 Chinese adults [18]. Their three-factor 16-item Stagnation Scale was subsequently validated with multiple samples, including students of university, adults in the community, patients with irritable bowel syndrome, and patients suffering from headaches as a consequence of the overuse of medication; and it was shown to have good, consistent psychometric properties [2, 19, 20]. After careful construct explication, the three factors were labeled body-mind obstruction, affect-posture inhibition, and overattachment. Body-mind obstruction involves somatic obstruction-like symptoms. Affect-posture inhibition involves being overly self-conscious and experiencing heightened awareness and uneasiness, which result in inhibited facial expressions and inhibited postural movements. Overattachment involves fear of losing what one possesses, being less accomplished than one desires to be and being unable to let go of some clinging.

Mindful of this factor structure, we designed a six-session body-mind-spirit (BMS) group therapy intervention for patients with stagnation syndrome [21]. An integrated BMS approach, grounded on TCM core beliefs and principles, was adopted as the theoretical framework for the group therapy [22]. An integrated BMS approach has been extensively applied in the treatment of a range of health and mental health conditions, including cancer, bereavement, depression, and infertility [18, 23–25]. The group therapy sessions are designed to be led by mental health practitioners after a brief training period (they do not need to have extensive prior knowledge of TCM). Further details of the BMS group therapy intervention are presented in the next section.

Pilot trials of our six-session BMS group therapy for stagnation syndrome had promising results [21, 26]. The aim of the present study was to more rigorously evaluate the efficacy of the intervention.

## 2. Methods

### 2.1. Study Design

A nonblind, two-arm randomized controlled trial (RCT) design was adopted: an intervention group received a six-session BMS intervention, and a control group received a six-session general TCM education course. For both groups, a two-hour session was held every week for six consecutive weeks. The participants were adult patients with stagnation syndrome. The trial was conducted from October 2014 to October 2015. The participants completed self-report scales and their salivary cortisol levels were measured at time T0 (when the pretest was administered); at time T1 (when the posttest was administered); and finally at time T2 (when the two-month follow-up assessment was conducted). The study was approved by the Institutional Review Board of the University of Hong Kong and the Hong Kong Hospital Authority (Ref. UW 13-137).

### 2.2. Participants

The participants were recruited from two TCM clinics (at different nongovernmental medical institutions) in Hong Kong. The inclusion criteria were (1) having a score of 50 or above on the Stagnation Scale [2]; (2) having been diagnosed as having stagnation syndrome (by a TCM practitioner) according to TCM criteria; (3) being aged 18 to 60; and (4) not having been diagnosed as having any life-threatening medical condition, such as cancer. All the participants provided their informed written consent before participating in the study.

For the recruitment, a 100% consecutive sampling scheme was adopted. An estimate of the effect size derived from a pilot study indicated that a sample size of 46 per arm was needed to achieve a power of 0.9. A trained research assistant was stationed at the TCM clinics during the predesignated recruitment periods. All patients met the inclusion criteria were invited to participate in the study. A hundred and thirty-five patients were assessed as eligible, and 126 of them agreed to participate in the study and provided with informed written consent. The participants were then randomly allocated, in equal numbers, to either an intervention group or a control group (with the aid of a random numbers table). Fifteen participants withdrew the study after randomization without completing any measures, with seven members in the intervention group and eight in the control group. Thus, the effective sample size of the study was 111 (56 in the intervention group and 55 in the control group).

**Figure 1:**
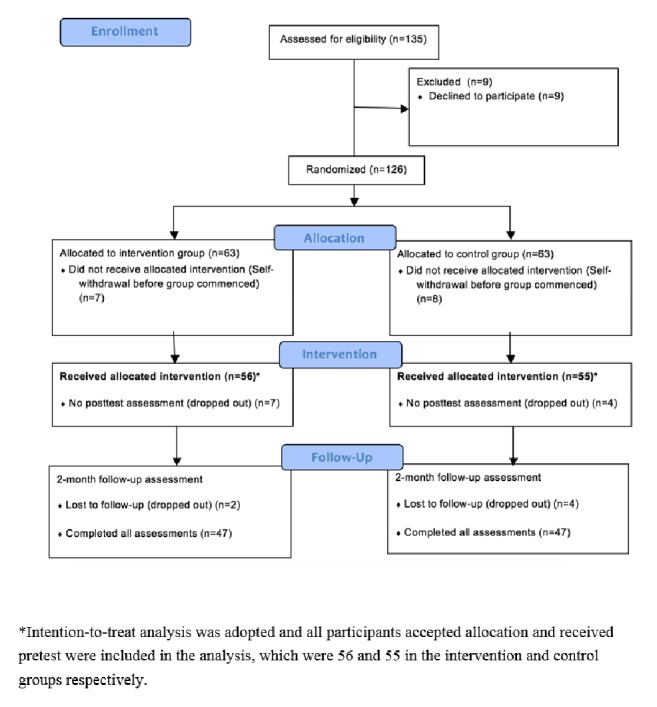
CONSORT diagram of participants' recruitment and completion numbers.

Because there is a capacity limit that each group may take, four pairs of intervention-control subgroups were formed (with an average of about 14 participants per group). The number of participants who completed the posttest and the two-month follow-up assessment was 49 and 47 members of the four intervention subgroups and 51 and 47 members of the four control subgroups; 87.5% of the intervention group completed the posttest and 84.0% completed the two-month follow-up assessment; 92.7% of the control group completed the posttest and 85.5% completed the two-month follow-up assessment.  Figure 1 is a flowchart showing the numbers of participants recruited for and of those who completed the RCT.

### 2.3. Intervention and Control Groups

#### 2.3.1. Intervention Group: Brief BMS Group Therapy Intervention for Patients with Stagnation Syndrome

The BMS group therapy for stagnation provided a two-hour manualized group therapy session once weekly for six consecutive weeks. The first two sessions focused on body-mind obstruction, with the aim of introducing the participating patients to self-administered TCM acupressure and health exercises for the rapid relief of symptoms. The third and fourth sessions focused on affect-posture inhibition, with the aim of enhancing the participants' awareness of excessive self-inhibition and fostering more adaptive behavior through group discussion and practice. The final two sessions focused on overattachment; the participants examined existential and spiritual issues in various group activities. For example, each member was asked to identify another group member who possessed similar beliefs about life; pairs or trios of these group members were then given extensive opportunities to discuss life issues (one of the tasks involved writing letters to each other). Through the group activities, each member gained personal insights and adjusted individual's beliefs/priorities in life.

To enhance treatment fidelity, the intervention protocol was manualized. The protocol has not been published, but it is available upon request by mental health researchers. For the present study, the intervention subgroups were led by the first and second authors of this paper (the first author has dual professional backgrounds in mental health social work and Chinese medicine; the second author has professional background in counseling psychology).

#### 2.3.2. Control Group

To control the social gathering effects of the intervention group, the control group received a parallel six-week general instruction course in TCM. Because learning about TCM is appealing to patients who suffer from stagnation syndrome, delivering this instruction to the control group helped researchers achieve a satisfactory completion rate. The group leader was a registered TCM practitioner. The six-week course included instruction in (1) the basic philosophy and principles of TCM; it imparted (2) basic knowledge of herbal medicine and acupuncture; and it taught the participants (3) how to apply TCM in their daily lives (including how to incorporate mild herbal formulas every day in the recipes for their meals) in order to promote their general well-being.

### 2.4. Measures

Stagnation, measured by means of a self-report multi-item scale, was the primary outcome measure. Secondary outcomes were measured by means of a number of related health and mental health self-report scales; and salivary cortisol, a biological marker of stress, was also measured.

Stagnation was assessed by means of the Stagnation Scale [2, 18]. This is a 16-item scale with possible scores ranging from 16 to 160; a higher score indicates more severe stagnation symptoms. The scale has good internal consistency (in the present sample the Cronbach's alpha coefficient for the whole scale was 0.91, and Cronbach's alpha coefficients for the overattachment, body-mind obstruction, and affect-posture inhibition subscales were 0.88, 0.83, and 0.90, respectively). A cutoff point of 50 was used in screening for stagnation syndrome (with false-positive and false-negative rates of 26% and 23%, respectively) [2].

Depressive symptoms were assessed by means of the Chinese version of the Patient Health Questionnaire [27] (PHQ-9). This is a nine-item scale; the possible scores range from 0 to 27, with a higher score denoting more severe depression symptoms. The nine items correspond to the nine DSM-IV criteria of a major depressive episode. The internal consistency was satisfactory (Cronbach's alpha coefficient was 0.88 [27]; the Cronbach's alpha coefficient in the present sample was 0.76). With a cutoff point of 9, the sensitivity and specificity of PHQ-9 for identifying clinical depression in Chinese adults were 80% and 92%, respectively.

Anxiety symptoms were assessed by means of the Chinese version of the Hospital Anxiety and Depression Scale's anxiety subscale [28, 29] (HADS-A). The scale has seven items; the possible scores range from 0 to 21, with a higher score indicating more severe anxiety symptoms. Internal consistency was revealed to be satisfactory (Cronbach's alpha coefficient was 0.77, Leung [28]; the Cronbach's alpha coefficient in the present sample was 0.81). The cutoff points were 7 (scores of 0 to 7 were taken to indicate a normal level of anxiety); 10 (scores of 8 to 10 were taken to indicate mild anxiety); and 14 (scores of 11 to 14 were taken to indicate a moderate level of anxiety, and scores of 15 to 21 were taken to indicate severe anxiety).

Physical distress, functioning, and effect were measured using subscales of the Body-Mind-Spirit Well-Being Inventory [30] (BMSWBI). The subscales utilized in the study were (1) the physical distress subscale; (2) the daily functioning subscale; and (3) the positive affect and negative affect subscale.

The BMSWBI physical distress subscale was used to assess the levels of subjective distress (caused by multiple somatic symptoms) that the respondents had experienced during the previous week [30]. The subscale consists of 14 items on somatic symptoms; the possible scores range from 0 to 140, with a higher score indicating a higher level of physical distress. The scale exhibited satisfactory internal consistency in the present sample (Cronbach's alpha coefficient = 0.87).

The BMSWBI daily functioning subscale was used to measure the participants' evaluation of their everyday functioning during the previous week [30]. The subscale consists of 10 items that cover the respondents' energy levels, levels of concentration, and levels of work motivation. The total possible scores range from 0 to 100, with a higher score indicating a higher level of functioning. The scale exhibited satisfactory internal consistency in the present sample (Cronbach's alpha coefficient = 0.85).

The BMSWBI positive affect and negative affect subscale was used to measure the participants' positive and negative affect. The subscale comprises eight items on positive affect and 11 items on negative affect [30]. The possible scores range from 0 to 80 and from 0 to 110, respectively. A higher score denotes a higher level of the affect concerned. The scale exhibited satisfactory internal consistency in the present sample (Cronbach's alpha coefficient = 0.89).

Salivary cortisol was measured as a biological marker of hypothalamic-pituitary-adrenal axis neuroendocrine functioning. Measures of salivary cortisol are frequently used in research on stress [31]. Prior to the pretest (baseline assessment), the salivary collection protocol was explained to all the participants; and the correct use of the salivary collection tools was demonstrated by a trained research assistant. The participants were advised to avoid consuming food and engaging in strenuous exercise for at least 30 minutes prior to collecting their saliva samples. The participants were also given written instructions. Prior to each assessment, the participants were given a set of five cotton Salivette tubes (Sarstedt, Numbrecht, Germany); and the participants took their own saliva samples immediately upon waking, 45 minutes later, at 12:00, at 17:00, and at 21:00 [32]. The participants were instructed to freeze their Salivette tubes (in the freezer compartment of a refrigerator) before returning them to the research team. The returned samples were kept frozen (at -20°C) until the research team used an immunoassay kit (from Salimetrics Inc., Carlsbad, California, USA) to measure the free cortisol. The laboratory facilities of the Department of Clinical Oncology at The University of Hong Kong were used for the laboratory work.

### 2.5. Statistical Analysis

The demographic and baseline clinical characteristics of the intervention and control group participants were compared. Independent samples* t*-tests were conducted for the continuous variables and *χ*^2^ analyses were conducted for the categorical variables (except for gender Fisher's exact test was used).

Intervention effects were measured by both between-group and within-group changes. The overall effect was first evaluated according to interaction time x group effect using repeated measures of doubly multivariate analysis of variance (MANOVA) on all nine outcomes variables: stagnation, depression, anxiety, physical distress, daily functioning, negative affect, positive affect, mean cortisol, and cortisol slope. And then for each outcome measure, including three stagnation subscales, repeated-measures univariate analysis of variance (ANOVA) was conducted to assess the effects on each outcome variable, respectively. Partial eta-square (*η*^2^) values were computed to assess the effect sizes; values of 0.02, 0.13, and 0.26 suggested small, medium, and large effect sizes, respectively [33]. The within-group effects (T1 versus T0 and T2 versus T0) were calculated through a paired t-test for each outcome variables. The effect sizes were assessed by calculating Cohen's* d* values; values of 0.2, 0.5, and 0.8 were taken to suggest small, medium, and large effect sizes, respectively. A two-sided *α* level of 0.05 was considered statistically significant.

An intention-to-treat analysis was performed to handle missing data due to dropped-out or failure to complete full set of assessments. The principle of “last observation carried forward” was adopted. Thus data of all participants who completed the pretest (baseline measures) were included in the data analysis, irrespective of whether these participants completed the posttest and two-month follow-up assessment or not.

The cortisol data were summarized by calculating the mean cortisol levels [34]; due to the skewed distribution of the cortisol data, natural logarithms were used to transform the raw cortisol data when calculating these mean values. The cortisol mean values at T0 (pretest), T1 (posttest), and T2 (2-month follow-up assessment) were subjected to the same interaction time x group effects and within-group effects analyses aforementioned. The diurnal cortisol rhythm (cortisol slope) was calculated by regressing log-transformed cortisol on collection time in linear regression.

## 3. Results

### 3.1. Participants Characteristics and Baseline Comparison

Table 1 summarizes the demographic characteristics and baseline measures of the 111 participants. There were no significant between-group differences found in the sociodemographic variables and no baseline differences observed in the nine outcome measures.

The mean age of the participants was 48.7 years (*SD* = 8.3). Eighty-one percent of the participants were female (80.4% in the intervention group; 81.8% in the control group). Over half of the participants were married or cohabiting (64.0% in total; 60.7% in the intervention group; 67.3% in the control group); working full-time (56.8% in total; 60.7% in the intervention group; 52.7% in the control group); and having a college level of education or above (52.3% in total; 51.8% in the intervention group; 52.7% in the control group).

The average scores for stagnation in the intervention and control group were 85.2 (*SD* = 25.2) and 78.1(SD=26.8), respectively, suggesting moderately severe level of stagnation. The mean PHQ-9 scores of both groups were higher than the cutoff point eight [11.0(SD=4.4) in the intervention group; 10.3(SD=5.1) in the control group].

### 3.2. Efficacy of Intervention

Table 2 summarizes the results of repeated-measures multivariate and univariate ANOVA of baseline assessment (T0), posttest (T1), and 2-month follow-up assessment (T2) of intervention and control groups. Firstly, combined effect was assessed by repeated measures of doubly MANOVA on all the nine outcome variables. The result suggested significant combined effect (*p*<0.001, *η*^2^= 0.42). The repeated-measures ANOVA revealed that the intervention group showed significant greater improvements in stagnation, the primary outcome, with medium effect size (p<0.01, *η*^2^= 0.11). The intervention group demonstrated significant greater decrease in three subscales of stagnation: body-mind obstruction (*p*<0.001, *η*^2^= 0.12), affect-posture inhibition (*p*<0.01, *η*^2^= 0.08), and overattachment (*p*<0.05, *η*^2^= 0.07). For secondary outcomes, intervention group also resulted in significantly greater reduction in depression (*p*<0.01, *η*^2^= 0.08), physical distress (*p*<0.001, *η*^2^= 0.13), everyday functioning (*p*<0.05, *η*^2^= 0.06), and negative affect (*p*<0.05, *η*^2^= 0.06). The intervention group did not show significantly greater improvements over control group in anxiety, positive affect, mean cortisol and cortisol slope.

Post hoc analysis revealed that the intervention group showed significantly greater improvements over the control group in cortisol level at two-month follow-up assessment (T0 versus T2) with small effect size (p<0.05, *η*^2^= 0.05), but not at posttest (T0 versus T1).

**Figure 2:**
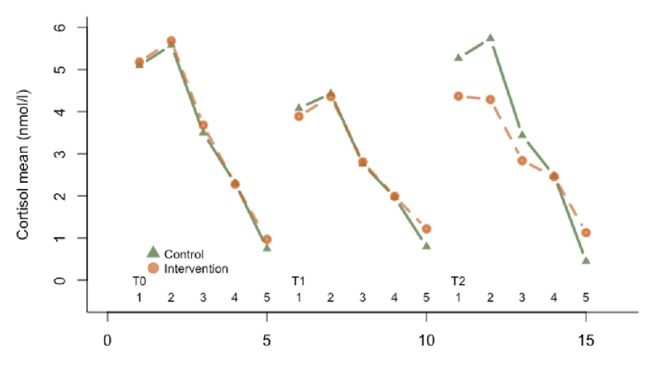
Mean diurnal salivary cortisol (nmol/l) at five times of day of participants in the intervention and control groups at pretest (T0), posttest (T1), and 2-month follow-up (T2).

Table 3 summarizes the results of the analysis of the within-group effects in the intervention and control groups. At both posttest and two-month follow-up, the intervention group showed significant improvement over baseline on all outcome measures, except for cortisol slope. Whereas the control group exhibited significant improvements over baseline at the posttest on two measures only: physical distress and mean cortisol levels. In the 2-month follow-up assessment, the control group showed significant improvements over baseline in stagnation, depression, anxiety, and negative affect as well. But the changes in the participants' mean cortisol became nonsignificant at this time point.  Figure 2 illustrates the diurnal pattern of cortisol level of participants in the intervention and control groups at pretest, posttest, and 2-month follow-up.

## 4. Discussion

Stagnation syndrome is a mind-body health condition; the term (and its culturally-specific treatment) originated in the TCM of ancient China. Our previous studies have operationalized the concept of stagnation syndrome into a construct useful to all mental health practitioners. While TCM has long-established physical treatments for the relief of the symptoms, there has not to date been a comprehensive mind-body intervention specifically for stagnation syndrome. To the best of our knowledge, the present study represents the first rigorous attempt at developing and evaluating a brief BMS group therapy intervention for patients with stagnation syndrome.

Overall, the results support the efficacy of the six-session BMS group intervention. Repeated measures of doubly MANOVA revealed a significant combined effect, with eta-squared at 0.42, which is in the range of large effect size. Repeated-measures ANOVA revealed that the intervention group showed significant improvements in stagnation, the primary outcome, with medium effect size (eta-squared = 0.11). The intervention group also showed significant improvements in depression, physical distress, everyday functioning, and negative affect (eta-squared = 0.06 to 0.13).

With respect to the within-group effects, the intervention group demonstrated significant improvements (against their baseline assessments)—in 11 measures—in both the posttest and the 2-month follow-up test. The effect size of the changes in stagnation, the primary outcome measure, was large. Significant changes in the intervention group's mean cortisol levels (a biological marker of stress) were also consistently observed at both time points and with medium effect sizes. A closer examination of the three component factors of stagnation reveals that the improvement in body-mind obstruction was the largest (Cohen's* d *ranging from 0.82 to 1.04). This is consistent with clinical experience: it is usually easier to relieve physical symptoms than to address the underlying psychosocial issues. Long-established, simple, self-administered TCM acupressure and traditional health exercises seemed to result in the rapid relief of the intervention group members' symptoms [35].

The observed improvements across a wide range of mind-body measures are congruent with the findings of previous empirical studies. Multidimensional positive benefits have previously been reported in mind-body interventions for depression [36, 37], anxiety [38], and tension headaches [39].

With regard to the interaction time x group effects, the efficacy of the BMS intervention revealed by the posttest results (T1) was impressive, with significant improvements in nine of the measures; these included stagnation, the primary outcome measure. The efficacy of the intervention looked less impressive in the results of the 2-month follow-up assessment (T2), with significant improvements evident in three measures only. However, a careful examination of the within-group effects suggests that the apparently reduced efficacy was not a result of diminished therapeutic effects of the BMS intervention but because of a moderate improvement in the general well-being of the members of the control group.

The daily use of mild herbal ingredients in their meals was a key content in the control group and aroused much interest among the participants. Such use of mild herbal/dietary ingredients might have certain nonspecific therapeutic effects in gradually enhancing overall well-being. This seems a plausible explanation of some of the improvements observed in the results of the control group's 2-month follow-up test.

Interestingly, the members of the control group showed significant improvement in their mean cortisol levels in the posttest but not in the 2-month follow-up assessment. Again, a possible explanation may be their daily use of the mild herbal-dietary recipes, which may have had some transient impacts on their hormonal systems. However, in-depth research in this area is called for. Over the long term, psychosocial factors significantly affect individuals' cortisol levels. This may explain the nonsignificant results in the control group members' mean cortisol levels in the 2-month follow-up assessment. In contrast, the improvement in the intervention group members' mean cortisol levels was well maintained in both the posttest and the 2-month follow-up assessment. This might be contributed by the overall reduction in distress level in participants in the intervention group.

The intervention demonstrated effectiveness in reducing participants' depression and enhancing everyday functioning 2 months after the treatment ended. According to the results, the interaction time x group effect became nonsignificant for stagnation but remained significant for depression and everyday functioning. On the one hand, a key reason for this was that the control group reported at T2 a bigger improvement in stagnation than in depression. A plausible explanation is that stagnation syndrome has a prominent somatic component and is thus likely to be more responsive to the mild herbal-dietary recipes used by the members of the control group. On the other hand, being focused on their physical health, the control group seemed to experience less amelioration of depression than did the members of the BMS intervention group; for the latter, the intervention not only focused on physical domain, but also put emphasis on psychosocial/cognitive changes. There are two possible mechanisms that might have occurred in the intervention process that brought about the positive change in depression. First of all, the intervention might help reduce participants' level of depression by enhancing their metacognitive efficiency and breaking the ruminative pattern of thinking. Previous research has consistently demonstrated the significant relationship between depression, rumination, and metacognition [40]. One of the key agendas of the intervention was to help participants expand their self-awareness with regard to maladaptive and repetitive process and patterns of perceiving, viewing, and thinking. With enhanced ability of being mindful to one's thinking process, participants might be able to reduce their rumination, which in turn contributed to reducing the level of depression. This might be why that overattachment, one of the three subscales in Stagnation Scale that resembles the concept of rumination, maintained significant time x group effect at T2. Secondly, the intervention might help participants reduce their level of depression by cultivating and nurturing a dynamic state of balancing. Dynamic balancing is an important conception in the BMS framework and a key element in the intervention (Lee et al., 2018). Similar to the concept of psychological flexibility, it encourages adaptive coping to changing and fluctuating situations, flexibility in shifting perspectives, and the way how one relates to himself/herself or others, and the acceptance and integration of negative experiences [41]. The cultivation of dynamic balancing might help participants accept the negative experiences relating to stagnation syndrome and develop adaptive copings, thus reducing the depression level. Consistently, McCracken and Gutiérrez-Martínez [42] also found that positive changes in psychological flexibility, brought about by acceptance and commitment therapy, were significantly related to reduced level of depression among individuals with chronic pain.

In contrast to mean cortisol, no significant changes in cortisol slope were observed in both the intervention and control groups. While both mean cortisol and cortisol slope are biomarkers of stress, cortisol slope is more reflective of the reactivity of the hypothalamic-pituitary-adrenal (HPA) axis activity [31]. Since the reactive pattern of HPA may not be able to change within a relatively short period of time, this could explain the lack of intervention effect on the cortisol slope. Further research with longer intervention and more follow-up assessments may help understand the impacts of the intervention on the neuroendocrine functioning of participants.

It is also worth noting the high completion rate for the intervention group: 88% completed the posttest and 84% completed the 2-month follow-up test (including collecting their saliva samples as instructed). High completion rates seem to suggest good acceptability and that people suffering from stagnation syndrome generally have a good experience of our brief BMS group therapy intervention.

Nevertheless, the present study has a number of limitations. First, the RCT had no blinding. However, in RCTs of psychosocial group interventions, it is difficult to completely blind the participants or therapists. Second, the basic TCM instruction provided to the control group seems to have thwarted the purpose of having a control group; the instruction seems to have brought about some nonspecific therapeutic effects. In our defense, when deciding to give this instruction to the control group a main consideration of ours was that if the members of the control group felt they had no reason to attend the meetings arranged for them then it would be hard to achieve a satisfactory completion rate. The members of the control group needed to have a substantial reason to attend the meetings in order to ensure good attendance rates. In future, we may test other control group designs. Third, for practical reasons the follow-up assessment was conducted only 2 months after the intervention ended. Because stagnation syndrome tends to be a chronic, persistent condition, it would have been desirable to have a longer follow-up period that would have helped us to gain a better understanding of the trajectory of the outcomes of our intervention.

To conclude, the results of the present study provide empirical evidence that indicates that our brief BMS group therapy intervention for patients with stagnation syndrome is efficacious. It would be worth repeating the trial in the context of other cultures and trying out other study designs (with appropriate control groups and/or comparison groups and with longer follow-up periods).

## Figures and Tables

**Table 1:** Baseline demographic characteristics and assessments–intervention and control groups.

Variables	Intervention Group (*n*=56)		Control Group(*n*=55)		*P *value^*a*^
	Mean (*SD*)	*n* (%)	Mean (*SD*)	*n* (%)	
**Age (Years) **	48.5(8.7)		48.9(8.1)		.45
**Gender**					.62
Female		45(80.4%)		45(81.8%)	
Male		11(19.6%)		10(18.2%)	
**Marital Status**					.72
Single		15(26.8%)		10(18.2%)	
Married/cohabiting		34(60.7%)		37(67.3%)	
Divorced/separated		4(7.1%)		4(7.3%)	
Widow		3(5.4%)		4(7.2%)	
**Employment**					.18
Full-time		34(60.7%)		29(52.7%)	
Part-time		3(5.4%)		6(10.9%)	
Retired		6(10.7%)		3(5.5%)	
Homemaker		11(19.6%)		12(21.8%)	
Unemployed		2(3.6%)		5(9.1%)	
**Education Level**					.27
Primary school		2(3.6%)		2(3.6%)	
Middle school		13(23.2%)		7(12.7%)	
High school		12(21.4%)		17(30.9%)	
College or above		29(51.8%)		29(52.7%)	
**Income (HK$)**_ _^**b**^					.63
None		14(25%)		14(25.5%)	
<$10,000		5(8.9%)		8(14.5%)	
$10,000-$19,999		11(19.6%)		8(14.5%)	
$20,000-$39,999		12(21.5%)		13(23.7%)	
≥ $40,000		14(25%)		12(21.8%)	

**Stagnation Total **	85.2(23.5)		78.1(26.8)		.27
**Body-mind Obstruction**	26.2(9.4)		24.2(10.2)		.32
**Affect-posture Inhibition**	18.0(7.6)		17.5(7.8)		.95
**Overattachment**	40.1(12.3)		38.2(13.6)		.38
**Depression **	11.0(4.4)		10.3(5.1)		.55
**Anxiety**	9.1(3.0)		10.1(4.1)		.19
**Physical Distress**	55.3(25.2)		56.7(25.7)		.62
**Everyday Functioning**	47.7(11.1)		46.4(13.6)		.51
**Negative Affect**	49.2(21.3)		50.5(19.3)		.59
**Positive Affect**	36.5(11.1)		36.1(15.4)		.88
**Mean Cortisol **(log nmol/L)	3.8(2.0)		3.4(1.4)		.51
**Cortisol Slopes**	-0.15 (0.10)		-0.16 (0.10)		.90

^a^Independent samples *t*-tests were conducted for the continuous variables, and *χ*^2^analysis was performed for the categorical variables, except for gender Fisher's exact test was used.  ^b^1US$ = 7.8HK$.

**Table 2:** Repeated-measures multivariate and univariate ANOVA of baseline assessment (T0), posttest (T1), and 2-month follow-up assessment (T2) of intervention and control groups (*N*=111).

Outcome variables	Overall efficacy (T0,T1 vs. T2)			Intervention efficacy (T0 vs. T1)			Maintenance effects (T0 vs. T2)		
	F(df)	Time×group (*p* value)	partial *η*^2^	F(df)	Time×group (*p* value)	partial *η*^2^	F(*df*)	Time×group (*p* value)	partial *η*^2^
**Combined Effect**_ _^a^ **Self-report Scales**	2.7(18)	0.00_ _^*∗∗∗*^	0.42						
Stagnation Total	6.3(108)	0.00_ _^*∗∗*^	0.11	11.6(109)	0.00_ _^*∗∗∗*^	0.97	2.7(109)	0.10	0.02
Body-mind Obstruction	7.2(108)	0.00_ _^*∗∗∗*^	0.12	14.3(109)	0.00_ _^*∗∗∗*^	0.13	1.6(109)	0.21	0.02
Affect-posture Inhibition	4.8(108)	0.01_ _^*∗∗*^	0.08	7.1(109)	0.01_ _^*∗∗*^	0.07	0.0(109)	0.98	0.00
Overattachment	4.1(108)	0.02_ _^*∗*^	0.07	9.9(109)	0.01_ _^*∗∗*^	0.08	3.6(109)	0.06	0.03
Depression	4.6(108)	0.01_ _^*∗∗*^	0.08	8.7(109)	0.00_ _^*∗∗*^	0.09	6.3(109)	0.01_ _^*∗*^	0.06
Anxiety	2.6(108)	0.08	0.05	4.2(109)	0.04_ _^*∗*^	0.04	0.3(109)	0.60	0.00
Physical Distress	8.1(108)	0.00_ _^*∗∗∗*^	0.13	9.6(109)	0.00_ _^*∗∗*^	0.10	0.1(109)	0.76	0.00
Everyday Functioning	3.3(108)	0.04_ _^*∗*^	0.06	5.3(109)	0.02_ _^*∗*^	0.05	5.2(109)	0.03_ _^*∗*^	0.05
Negative Affect	3.4(108)	0.04_ _^*∗*^	0.06	6.4(109)	0.01_ _^*∗∗*^	0.07	1.3(109)	0.25	0.01
Positive Affect	1.1(108)	0.34	0.02	1.0(109)	0.31	0.01	1.0(109)	0.14	0.02
**Salivary Cortisol**									
Mean Cortisol (log nmol/L)	1.6 (108)	0.21	0.03	0.9(109)	0.35	0.01	4.7(109)	0.03_ _^*∗*^	0.05
Cortisol Slope	1.0. (108)	0.39	.02	0.9(109)	0.36	0.01	1.4(109)	.0.23	0.01

^a^Repeated measures of doubly MANOVA on all 9 outcome variables, that are stagnation total, depression, anxiety, physical distress, everyday functioning, negative affect, positive affect, mean cortisol, and cortisol slope.

^*∗*^ *p* ≤ .05. ^*∗∗*^*p* ≤ .01. ^*∗∗∗*^*p* ≤.001.

**Table 3:** Within-group comparison of outcomes: baseline assessment, posttest, and 2-month follow-up assessment.

	Intervention Group (*n*=56)	Baseline Assessment(T0)	Posttest (T1)		Two-month Follow-up Assessment (T2)	
	Control Group (*n*=55)	Mean (*SD*)	Mean (*SD*)	Effect size (*d*)	Mean (*SD*)	Effect size (*d*)
**Self-report Scales**	**Stagnation Total**					
	Intervention group	85.2(23.5)	64.3(22.7)_ _^*∗∗∗*^	-0.90	66.9(23.6)_ _^*∗∗∗*^	-0.78
	Control group	78.1(26.8)	73.0(26.7)	-0.19	67.9(24.6)_ _^*∗∗*^	-0.40
	**Body-mind Obstruction**					
	Intervention group	26.2(9.4)	17.2(7.8)_ _^*∗∗∗*^	-1.04	18.7(8.8)_ _^*∗∗∗*^	-0.82
	Control group	24.2(10.2)	22.1(9.8)	-0.21	19.7(8.1)_ _^*∗∗∗*^	-0.47
	**Affect-posture Inhibition**					
	Intervention group	18.0(7.6)	13.4(6.5)_ _^*∗∗∗*^	-0.65	14.9(7.4)_ _^*∗∗*^	-0.41
	Control group	17.5(7.8)	16.6(7.6)	-0.11	15.1(6.9)_ _^*∗*^	-0.33
	**Overattachment**					
	Intervention group	40.1(12.3)	30.3(10.5)_ _^*∗∗∗*^	-0.86	30.7(10.5)_ _^*∗∗∗*^	-0.82
	Control group	38.2(13.6)	34.6(13.3)	-0.27	32.6(13.5)_ _^*∗∗*^	-0.41
	**Depression**					
	Intervention group	11.0(4.4)	7.8(3.7)_ _^*∗∗∗*^	-0.79	7.6(4.2)_ _^*∗∗∗*^	-0.79
	Control group	10.3(5.1)	9.4(4.9)	-0.18	8.9(5.0)_ _^*∗∗*^	-0.28
	**Anxiety**					
	Intervention group	9.1(3.0)	7.7(3.0)_ _^*∗∗*^	-0.47	7.6(3.2)_ _^*∗∗∗*^	-0.48
	Control group	10.1(4.1)	9.9(4.4)	-0.05	8.7(3.7)_ _^*∗∗*^	-0.36
	**Physical Distress**					
	Intervention group	55.3(25.2)	37.4(19.2)_ _^*∗∗∗*^	-0.80	43.8(25.2)_ _^*∗∗*^	-0.47
	Control group	56.7(25.7)	50.7(25.8)_ _^*∗*^	-0.23	46.7(24.0)_ _^*∗∗∗*^	-0.40
	**Everyday Functioning**					
	Intervention group	47.7(11.1)	53.9(12.5)_ _^*∗∗∗*^	0.52	54.5(12.7)_ _^*∗∗∗*^	0.57
	Control group	46.4(13.6)	47.4(13.1)	0.07	47.4(14.1)	0.07
	**Negative Affect**					
	Intervention group	49.2(21.3)	40.4(19.8)_ _^*∗∗*^	-0.43	39.4(21.3)_ _^*∗∗*^	-0.46
	Control group	50.5(19.3)	50.3(21.2)	-0.01	45.2(22.0)_ _^*∗*^	-0.26
	**Positive Affect**					
	Intervention group	36.5(11.1)	39.4(12.1)_ _^*∗*^	0.25	40.3(13.0)_ _^*∗*^	0.31
	Control group	36.1(15.4)	37.2(6.1)	0.09	36.9(17.6)	0.05
**Salivary Cortisol**	**Mean Cortisol (log nmol/L)**					
	Intervention group	3.6(2.0)	2.9(1.3)_ _^*∗∗*^	-0.56	3.0(1.3)_ _^*∗∗*^	-0.48
	Control group	3.4 (2.0)	2.8(1.5)_ _^*∗*^	-0.43	3.5(2.2)	0.03
	**Cortisol Slope**					
	Intervention group	-0.15 (0.10)	-0.17(0.11)	0.19	-0.13(0.10)	-0.19
	Control group	-0.15(0.10)	-0.18(0.11)	0.29	-0.18(0.21)	0.18

^*∗*^ *p *≤ .05. ^*∗∗*^*p *≤ .01. ^*∗∗∗*^*p *≤.001.

## Data Availability

The data used to support the findings of this study are available from the corresponding author upon request.
